# Modification of as Synthesized SBA-15 with Pt nanoparticles: Nanoconfinement Effects Give a Boost for Hydrogen Storage at Room Temperature

**DOI:** 10.1038/s41598-017-04346-9

**Published:** 2017-07-03

**Authors:** Yu Yin, Zhi-Feng Yang, Zhi-Hao Wen, Ai-Hua Yuan, Xiao-Qin Liu, Zhuang-Zhuang Zhang, Hu Zhou

**Affiliations:** 1 School of Environmental and Chemical Engineering, Jiangsu University of Science and Technology, Zhenjiang, 212003 China; 2 State Key Laboratory of Materials-Oriented Chemical Engineering, College of Chemistry and Chemical Engineering, Nanjing Tech University, Nanjing, 210009 China; 3 School of Material Science and Engineering, Jiangsu University of Science and Technology, Zhenjiang, 212003 China

## Abstract

In this work, Pt nanoparticles were incorporated into SBA-15 to prepare the materials for hydrogen spillover adsorption. We provide a direct modification (DM) strategy to improve the content of Pt nanoparticles inside the channels of SBA-15. In this strategy, the Pt precursor was directly incorporated into as synthesized SBA-15 by a solid-state grinding method. The subsequent calcination in air, then H_2_/Ar gases was conducted to obtain the resultant materials of PtAS. For the samples of PtAS, Pt nanoparticles up to 5.0 wt% have a high dispersion inside the channels of SBA-15. The size of nanoparticles is in control of 3.7 nm. Although much work so far has focused on modification of SBA-15 with Pt nanoparticles. Here, it is the first time the loading amount of Pt nanoparticles raises up to 5.0 wt%, and the location of the Pt nanoparticles is interior channels of SBA-15. We reveal that the high dispersion behaviors of Pt nanoparticles are ascribed to the nanoconfinement effects provided by as synthesized SBA-15. However, the samples derived from template free SBA-15 (PtCS) show sparsely dispersion of Pt nanoparticles with the size of 7.7 nm. We demonstrate that the PtAS samples show better hydrogen adsorption performance than PtCS.

## Introduction

With the vigorous development of new energy technology, significant attention has been paid to hydrogen gas because it is an environmental friendly source and has the potential application in power fuel cells^1–4^. Despite the advantages, the use of hydrogen as an energy source for transportation applications is subject to limitations, and one of the significant challenges is its efficient storage^5–9^. According to the targets of the US Department of Energy, to meet the demands for onboard vehicular applications, the storage capacity should be over 7.5 wt% at a temperature of 298 K and a maximum pressure of 12 bar^10^. Among various alternatives, hydrogen spillover is considered as one of the most promising technologies to achieve the targets, because it is capable of storage hydrogen under mild conditions with high capacity^11–18^. It is known that there are two main components working for the hydrogen spillover process. One is the metal particle, which assumes the role of chemisorption and dissociation of hydrogen molecules, and is defined as the active sites. The other is a support, and is described as the accepting surface, which is in charge of reception and diffusion of hydrogen atoms migrated from the active sites^19–23^. It is demonstrated that the dissociation of hydrogen source by the metal particle is an essential step, and then a support with high surface areas could work for the storage of hydrogen source. It is reasonable to believe that, the accepting surface surrounding the metal particle has more opportunity to contact with dissociated hydrogen atoms. However, the accepting surface far from the metal particle shows little chance to diffuse hydrogen atoms. Thus, the storage capacity is strongly dependent on the dispersion behaviors of metal particles on the support.

Mesoporous silica SBA-15 is widely studied in the fields of catalysis^24–26^, gas separation^27–30^, drug delivery^31, 32^, and energy storage^33, 34^. It is of great interest for use as a support to disperse heterogeneous noble metal nanoparticles, due to its ordered pore structure, high surface area and large pore volume^35–39^. So far, an enormous amount of research effort goes into modification of SBA-15 with noble metal nanoparticles. Incorporation of Pt nanoparticles into SBA-15 has been conducted by various methods^40^, including impregnation^41^, deposition-precipitation^42^, graft hybrid^43^, colloid immobilization^44^, vacuum calcination^45^ and so on. On the basis of these methods, Pt nanoparticles with suitable size could be successfully introduced into the internal channels of SBA-15. However, the reported content of Pt nanoparticles locating inside the channels of SBA-15 is always below 1.0 wt%. It should not be ignored that the allowed content of Pt nanoparticles locating inside the channels of SBA-15 is quite low. It remains challenging to develop an efficient method to modify SBA-15 with high content of Pt nanoparticles inside the channels. It is found that, in the reported methods, as synthesized SBA-15 is first to calcination to remove template and generate open pores. The Pt precursor is then incorporated into the template free SBA-15, followed by the calcination and reduction to generate Pt nanoparticles. It is reported by Zhu and Sun’s groups that there is an extraordinary confined space between the template and silica walls in as synthesized SBA-15^46, 47^. By use of such a space, Zhu’s group created a new kind of CO_2_ capture^48^, and Sun’s group achieved the monolayer dispersion degree of active metal oxides with high loading amount^49, 50^.

**Figure 1:**
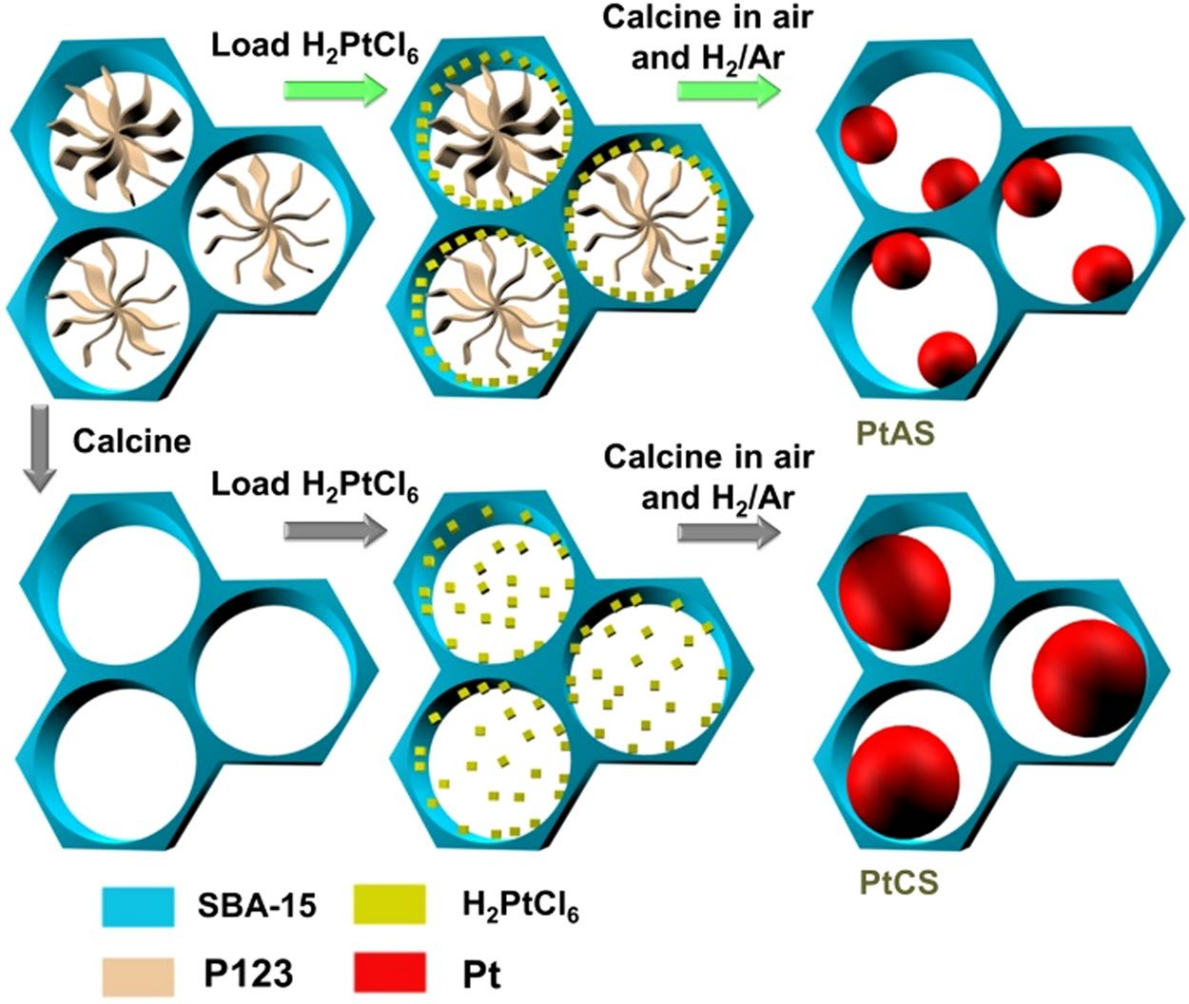
Formation of highly dispersed Pt nanoparticles (PtAS) by using the confined space provided by as synthesized SBA-15, and sparsely dispersed Pt nanoparticles (PtCS) with the template free SBA-15.

In this study, the Pt precursor was directly incorporated into as synthesized SBA-15 by a solid-state grinding method (Fig. 1). The subsequent calcination in air, then H_2_/Ar gases was conducted to obtain the resultant materials, which were denoted as *x*PtAS. The prefix letter points the content of Pt (wt%) in the resultant samples. According to the results, the Pt precursor could be drilled into the confined space in the solid-state grinding step. With the following calcination process, nanoconfinement effects would occur and work for the eventual formation of highly dispersed Pt nanoparticles inside the channels of SBA-15. We provide a direct modification (DM) strategy to prepare noble metal nanoparticles inside the channels of mesoporous silica. By use of such a DM strategy, 5.0 wt% of Pt nanoparticles are highly dispersed over the whole inner surface of SBA-15 with the particle size of 3.7 nm. This is the first time to have a report on the 5.0 wt% loading amount of Pt nanoparticles inside the channels of SBA-15. That is to say, our DM strategy endows a rather high loading amount of Pt nanoparticles inside the channels of mesoporous silica. Contributing to this DM strategy, active sites have a high dispersion on the accepting surface with a high loading content, which guarantees the internal surface of the support could efficiently play the role of accepting hydrogen source. The Pt precursor was also incorporated into template free SBA-15 to prepare counterpart samples of *x*PtCS. In the 5.0PtCS sample, the Pt nanoparticles are sparsely distributed on SBA-15 with the size of 7.7 nm. The larger particle size results in the fewer number of nanoparticles, while the incorporated amount is equal to 5.0 wt%. We also demonstrate that the obtained materials (PtAS) prepared with the DM strategy exhibit excellent performance on hydrogen storage at room temperature, which is evidently better than the materials derived from template free SBA-15 (PtCS). The uptake of hydrogen at 100 kPa reaches the high value of 0.42 mmol∙g^−1^ for the sample of 5.0PtAS. It overcomes the value of 0.21 mmol∙g^−1^ for the sample of 5.0PtCS.

## Results and Discussion

### Dispersion behaviors of the Pt nanoparticles

**Figure 2:**
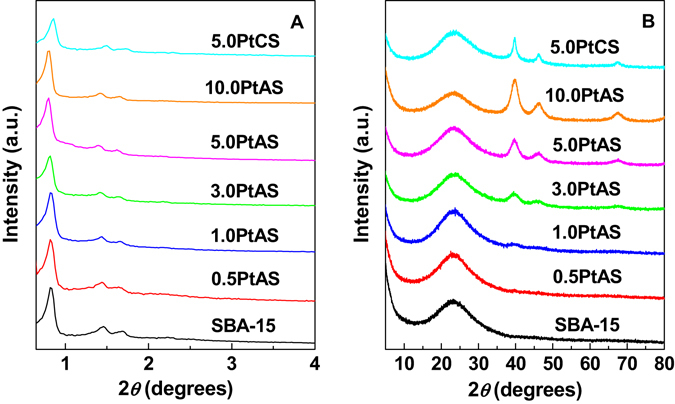
(**A**) Low-angle and (**B**) wide-angle XRD patterns for the samples of SBA-15, PtAS and PtCS.

Figure 2A shows the low-angle XRD patterns of the support SBA-15 and Pt modified samples. All samples show an intense diffraction peak accompanied by two weak peaks, which could be assignable to (100), (110) and (200) reflections of SBA-15. This indicates that the two-dimensional hexagonal pore symmetry is retained after the Pt modification. It could also be seen that, compared with the parent SBA-15, the *d*-spacings of Pt modified samples have a slight movement (Figure S1). The values of PtAS samples shift to higher points, however, that of PtCS sample shifts to a lower point. Further calculations reveal that the unit cell constant (a_0_) is 12.4 for the SBA-15, and 11.9 for 5.0PtCS. The a_0_ values of PtAS samples increase from 12.4 to 12.7, with the growing number of Pt content. It is known that the mesoporous frameworks show shrinkage at high temperature calcination. The PtCS sample with repeated calcination thus has a smaller unit cell constant in contrast to SBA-15. It is worth noting that, the PtAS samples show the same or even larger unit cell constants than SBA-15. This means the framework shrinkage is in control, probably due to the incorporation of Pt into as synthesized SBA-15.

Figure 2B depicts the wide-angle XRD patterns of all the samples. For the support SBA-15, there is a single broad diffraction peak centered at 23°, which is assignable to amorphous silica walls. For the samples of PtAS, three new diffraction peaks at 39.8°, 46.2° and 67.4° stemming from Pt (JCPDS no. 04–0802) appear. The three peaks correspond to the (111), (200), and (220) facets of Pt. This suggests that the Pt particles do exist in the resultant materials. Moreover, the diffraction peaks become intense with the increase of Pt content. The sample of 5.0PtCS prepared from template free SBA-15 also shows three Pt diffraction peaks. It should be noted that the Pt diffraction peaks of PtCS is much narrower than that of PtAS samples. The average sizes of the Pt particles were calculated by the Scherrer formula based on full-width at half maximum values from Pt(111) peaks, and the results are listed in Table 1. The particle sizes of Pt in the samples of PtAS are in the range of 1.6–3.7 nm, while Pt in the sample of 5.0PtCS exhibits a much larger particle size of 7.7 nm.

**Table 1:** Physicochemical properties of SBA-15, PtAS and PtCS samples.

Sample	S_BET_ (m^2^·g^−1^)	*V*_*p*_ (cm^3^·g^−1^)	*D*_*p*_ (nm)	a_0_^a^ (nm)	d_Pt_^b^ (nm)
SBA-15	710	1.227	9.2	12.4	—
0.5PtAS	738	1.222	9.2	12.4	1.6
1.0PtAS	751	1.244	9.2	12.4	2.1
3.0PtAS	739	1.233	9.2	12.4	3.1
5.0PtAS	728	1.217	8.9	12.7	3.7
10.0PtAS	743	0.836	6.8	12.7	3.7
5.0PtCS	595	0.847	8.2	11.9	7.7

^a^Unit cell constant calculated according to a_0_ = 2 × 3^−1/2^ × d_100_.

^b^Pt crystallite size calculated by the Scherrer formula.

**Figure 3:**
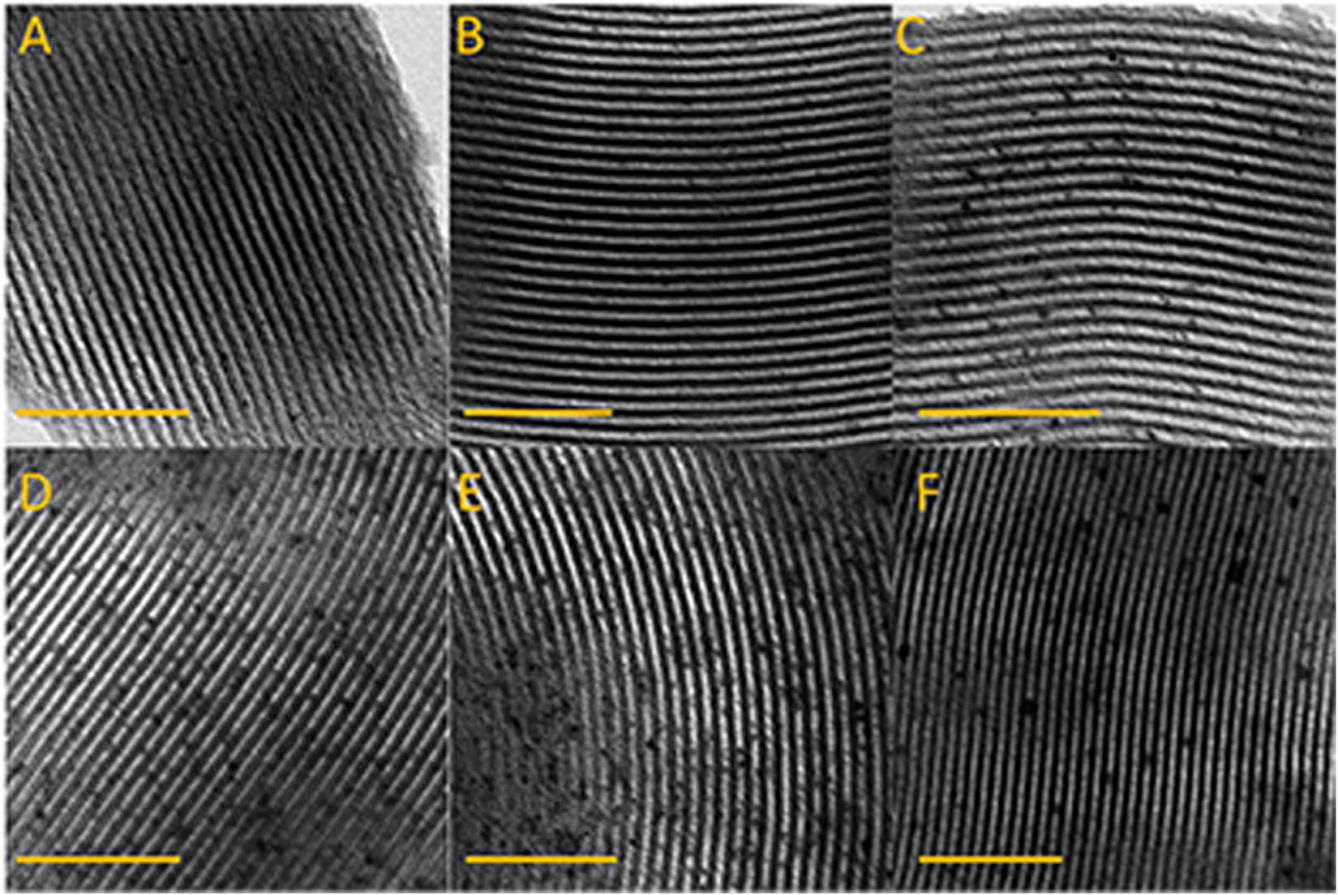
TEM images for the samples of (**A**) 0.5PtAS, (**B**) 1.0PtAS, (**C**) 3.0PtAS, (**D**) 5.0PtAS, (**E**) 10.0PtAS, and (**F**) 5.0PtCS. Scale bars represent 100 nm.

TEM is a powerful technique to characterize the structure of mesoporous materials modified with nanoparticles. The images of the samples of PtAS and PtCS are shown in Fig. 3. For all the samples, the ordered hexagonal arrays could be clearly observed. This indicates that the structure of SBA-15 is well preserved after the incorporation of Pt nanoparticles. Besides, Pt nanoparticles could be easily identified in the images. For the samples of PtAS with Pt content from 0.5 to 5.0 wt%, both the size and the number of the nanoparticles grow up with the increase of Pt content. In addition, the appropriate particle sizes allow the Pt nanoparticles locate inside the channels of SBA-15. It is found that there is a certain distance between the one with another nanoparticle. In other words, the nanoparticles have a high dispersion inside the channels of SBA-15. To further verify the relative positions of Pt nanoparticles and silica walls of SBA-15, HRTEM image for 5.0PtAS was obtained and the result was displayed in Figure S2. It is observed that Pt nanoparticles locate inside the channels of SBA-15 rather than other places. While the Pt content is up to 10.0 wt%, the sample of 10.0PtAS shows the similar Pt particle size with that of 5.0PtAS. Although the Pt nanoparticles locate inside the channels, the Pt content is so high that the space between the particles is very cramped. There is a sign that the channels are blocked by the densely dispersed Pt nanoparticles to some extent. Despite the identical Pt content with the sample of 5.0PtAS, the sample of 5.0PtCS derived from template free SBA-15 shows obviously different images. The large size of nanoparticles could be identified, which results in the few number of Pt nanoparticles. It is then found that a long range of channels without Pt nanoparticles modified on the surface.

**Figure 4:**
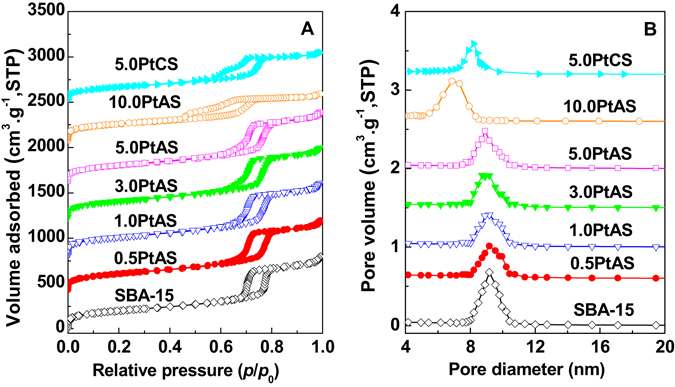
(**A**) N_2_ adsorption–desorption isotherms and (**B**) pore size distributions of SBA-15, PtAS, and PtCS samples. Curves are plotted offset for clarity.

Figure 4 displays the (A) N_2_ adsorption–desorption isotherms and (B) pore size distributions of samples. Surface areas, pore volumes, and pore diameters calculated according to the isotherms are listed in Table 1. Parent SBA-15 shows a type IV isotherm with an H1 hysteresis loop at the relative pressure of 0.68–0.78. This indicates that the template free SBA-15 has ordered cylindrical mesopores. The attendant pore diameter is 9.2 nm. For the samples of PtAS with Pt content from 0.5 to 5.0 wt%, the shapes of isotherms are similar with the parent SBA-15, except in the case of closing pressure of the hysteresis loops. The hysteresis loops for 0.5PtAS, 1.0PtAS, 3.0PtAS and 5.0PtAS samples close at the relative pressure of 0.60. The lower closing pressure of hysteresis loops than SBA-15 gives an evidence that the location of the nanoparticles is the internal channels of SBA-15. The pore diameters are 9.2 nm for the samples of 0.5PtAS, 1.0PtAS, and 3.0PtAS. The low content of the nanoparticles attached to the silica walls might be the reason for the unchanged pore diameters, and it is simply to cause the delay of desorption isotherms. While the Pt content is up to 5.0 wt%, as for the sample of 5.0PtAS, the decreasing of the pore diameter down to 8.9 nm is observed. The isotherms of 10.0PtAS differ from other series of PtAS samples. Desorption branch of the isotherm is apparently delayed. The hysteresis loop closes at the relative pressure of 0.45. The further reducing pore diameter of 6.8 nm is observed. According to the TEM images, although ultra-high Pt content, the Pt nanoparticles could still locate inside the channels of SBA-15 with high density. The distorted hysteresis loop and reduced pore diameter also confirm that the location of excess Pt nanoparticles is the internal channels. There has been a jump in the BET surface areas of PtAS samples (728–751 m^2^·g^−1^) compared with pure SBA-15 (710 m^2^·g^−1^). The increasing values should be provided by the surface areas of Pt nanoparticles. For the sample derived from template free SBA-15, 5.0PtCS shows that the hysteresis loop closes at the relative pressure of 0.55. The shift of the closing pressure of hysteresis loops comparing with SBA-15 indicates that the Pt nanoparticles are inside the channels. The pore diameter is 8.2 nm. The BET surface areas of 5.0PtCS sample (595 m^2^·g^−1^) are lower than SBA-15. The location and the large size of the Pt nanoparticles are the probable reasons for the decreasing pore diameter and surface areas. It is seen that the pore characteristics of 5.0PtCS are quite different from the 5.0PtAS sample, even if the Pt content is identical.

Based on the above description, it is conclusive that the sizes of obtained Pt nanoparticles are smaller than the pore diameters of mesoporous silica SBA-15. Thus the Pt nanoparticles in the samples should locate inside the channels rather than other places. And with the different preparation methods and loading content, the Pt nanoparticles show three states, namely Pt nanoparticles highly dispersed in the size of not exceeding 3.7 nm (for 0.5PtAS, 1.0PtAS, 3.0PtAS, and 5.0PtAS samples), Pt nanoparticles densely dispersed in the size of 3.7 nm (for 10.0PtAS sample), and Pt nanoparticles sparsely dispersed in the large size of 7.7 nm (for 5.0PtCS sample).

### Proposed mechanism for nanoconfiment effects

**Figure 5:**
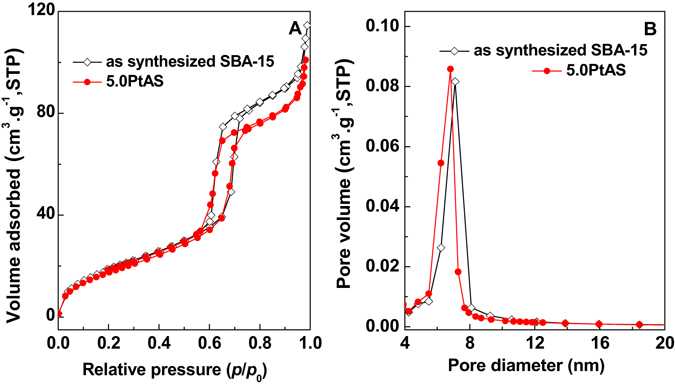
(**A**) N_2_ adsorption-desorption isotherms and (**B**) pore size distributions of as synthesized SBA-15, and 5.0PtAS samples before calcination.

According to the foregoing results, it is found that direct introduction of the Pt Precursor into the as synthesized SBA-15 promotes the dispersion degrees of Pt nanoparticles. The Pt nanoparticles are smaller in size for the PtAS samples than that of PtCS. The existence or non-existence of the template P123 is proposed as the main reason for the different dispersion behaviors. It was reported by the Zhu and Sun’s groups, there is a confined space between template and silica walls in as synthesized SBA-15^46, 47^. And our results from the N_2_ sorption isotherms of the as synthesized samples with no calcination could provide direct evidences for the existence of such a confined space. As shown in Fig. 5, some uptake and hysteresis could be observed in the N_2_ sorption isotherm of as synthesized SBA-15. This properly illustrates the existence of a confined space in as synthesized SBA-15. In addition, the incorporation of the Pt precursor into this confined space could be verified. The space volume of the as synthesized SBA-15 is 0.177 cm^3^·g^−1^ (Table 2). For the sample of freshly ground 5.0PtAS, the space volume decreases to 0.156 cm^3^·g^−1^. According to the experimental process, the ratio of the Pt precursor in the freshly ground 5.0PtAS is 6.2 wt%. That is to say, the ratio of as synthesized SBA-15 in the freshly ground 5.0 PtAS is 93.8 wt%, which could provide a space volume of 0.177 × 0.938 = 0.166 cm^3^·g^−1^. However, the measured value (0.156 cm^3^·g^−1^) is much lower. The best explanation is that the introduced 6.2 wt% of Pt precursor drill into the confined space in 93.8 wt% of as synthesized SBA-15.

**Table 2:** Physicochemical properties of as synthesized SBA-15, and 5.0 PtAS samples before calcination.

Sample	S_BET_ (m^2^·g^−1^)	*V*_*p*_ (cm^3^·g^−1^)	*D*_*p*_ (nm)
as synthesized SBA-15	73	0.177	7.1
as synthesized 5.0PtAS	69	0.156	6.8

**Figure 6:**
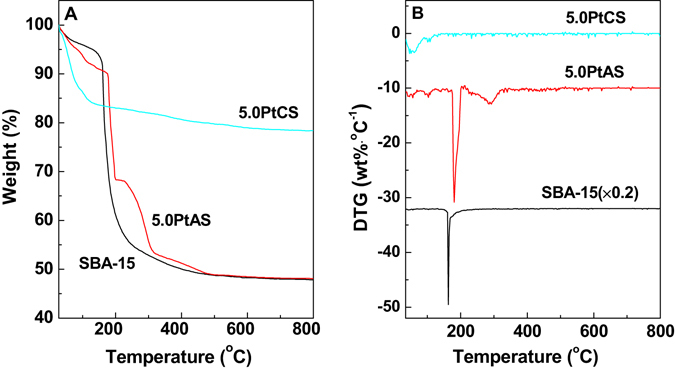
(**A**) TG and (**B**) DTG curves of SBA-15, 5.0PtAS and 5.0PtCS samples before calcination. DTG curves are plotted offset for clarity.

TG is a useful technique to investigate the decomposition procedures of different samples. As profiled in Fig. 6, the decomposition of the 5.0PtCS sample starts with a removal of water, in the temperature range of 30 to 100 °C. The corresponding weight loss is 14.0%. It subsequently emerges a continuous weight loss of 7.5%, from 100 to 800 °C, which is attributed to the conversion of H_2_PtCl_6_. In as synthesized SBA-15, the decomposition of template P123 goes from 155 to 300 °C, with a sharp weight loss of 43.1%, corresponding to the DTG peak at 162 °C. The gradual weight loss of 4.8% from 300 to 800 °C should be contributed to the removal of residual carbonaceous species. It is reported that the decomposition temperature of pure P123 is 210 °C^51^. The lower decomposition temperature of P123 in as synthesized SBA-15 (162 °C) is profited from the catalysis of the silica frameworks. For the 5.0PtAS sample, the conversion of H_2_PtCl_6_ proceeds from 100 to 177 °C with a weight loss of 4.0%. Afterwards, the decomposition of template goes in three steps: the first weight loss of 21.4% between 177 to 198 °C, the second weight loss of 14.7% between 198 to 312 °C, and the third loss of 5.5% between 312 to 500 °C. The sharp DTG peak at 182 °C reveals an interesting rule. Whichever step, the decomposition temperature of P123 in 5.0PtAS is higher than in as synthesized SBA-15. This confirms the successful introduction of the Pt precursor into the confined space in synthesized SBA-15, which separates the template from the silica frameworks to some extent. As a result, the catalysis efficiency of the silica frameworks has a significant reduction. It should be stated that the conversion sequence of the Pt precursor and template has an important effect on the resultant dispersion behaviors of Pt nanoparticles. If the conversion of the Pt precursor occurs before the removal of template, the confined space could work for the process of H_2_PtCl_6_ decomposition, which could control the Pt nanoparticles in a small size. While the template is removed prior to the conversion of the Pt precursor, the Pt nanoparticles will grow into a large size, due to the destruction of the confined space.

**Figure 7:**
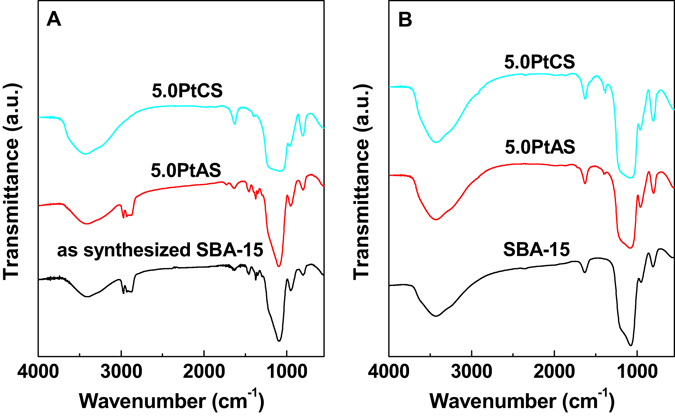
IR spectra of SBA-15, 5.0PtAS and 5.0PtCS samples (**A**) before and (**B**) after calcination.

Although there is a remaining weight loss above the temperature of 300 °C in the sample of 5.0PtAS. The decomposition of P123 is further studied with IR profiles (Fig. 7) and elemental analysis results (Table 3). The IR bands in the ranges 2850–3000 and 1350–1500 cm^−1^ are the characteristics of P123 in the samples of as synthesized SBA-15 and 5.0PtAS^47, 52^. After calcination, the bands of P123 become invisible. It is reasonable to infer that the template has a massive decomposition. For the sample of 5.0PtCS, whether calcination or not, there is no bands of P123 present. The elemental analysis results show that there is 30.59 wt% of C in as synthesized SBA-15. With the incorporation of the Pt precursor, the ratio of C decreases to 27.87 wt% for the freshly ground 5.0PtAS sample. After calcination with increasing temperatures, the ratio of C gradually decreases. With calcination at 300 °C for 2 h, the ratio of C is down to 1.817 wt%. It could be considered that the majority of P123 has been decomposed.

**Table 3:** Elemental analysis results of C and H weight ratio in the samples.

sample	C (wt %)	H (wt %)
as synthesized SBA-15	30.59	5.83
as synthesized 5.0PtAS	27.87	5.56
5.0PtAS-150	19.13	4.13
5.0PtAS-200	6.691	3.37
5.0PtAS-250	3.052	3.14
5.0PtAS-300	1.817	2.95

In summary, nanoconfinement effects working for the high dispersion of Pt nanoparticles depends on three key requirements: (i) there is an extra confined space in as synthesized SBA-15, (ii) the Pt precursor is incorporated into the confined space between template and silica walls, (iii) the conversion of the Pt precursor occurs before the removal of template. It is coincident that the PtAS samples meet the three requirements. Thus the high dispersion of Pt nanoparticles inside the channels of SBA-15 is achieved.

### Hydrogen spillover performance

**Figure 8:**
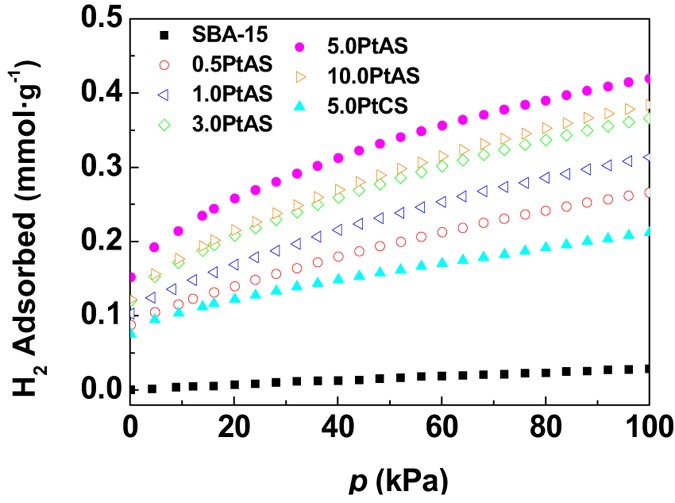
Hydrogen adsorption isotherms at 298 k for the samples of SBA-15, PtAS, and PtCS.

Figure 8 presents the hydrogen adsorption isotherms of the resultant materials at 298 K. The support SBA-15 shows a negligible adsorption capacity of 0.03 mmol∙g^−1^ at 100 kPa. The modification of SBA-15 with Pt nanoparticles improves the adsorption capacity evidently. This should be reasonably attributed to the hydrogen spillover mechanism. The hydrogen source firstly contacts with the active Pt nanoparticles, and is dissociated to hydrogen atoms. The surface of the SBA-15 surrounding the Pt nanoparticles is then capable to accept hydrogen atoms. The results show that the dispersion behaviors of the Pt nanoparticles play an important role on the adsorption performance. For the samples of 0.5PtAS, 1.0PtAS, 3.0PtAS and 5.0PtAS, the Pt nanoparticles are highly dispersed inside the channels of SBA-15 attributed to nanoconfinement effects. That is to say, there is a certain undecorated inner surface of mesoporous silica surrounding each nanoparticle. In these four samples, the amount of hydrogen captured at 100 kPa steps up from 0.27 to 0.42 mmol∙g^−1^ with the increase of Pt content, and the highest value is 0.42 mmol∙g^−1^ for the sample of 5.0PtAS. As for the sample of 10.0 PtAS, the adsorption capacity is 0.38 mmol∙g^−1^ at 100 kPa. Too densely dispersed Pt nanoparticles, slightly blocked pores, and less undecorated inner surface should make the compromise of nanoconfinement effects. The counterpart sample of 5.0PtCS shows the worst performance among all the Pt modified samples. It is capable of adsorbing 0.21 mmol∙g^−1^ of hydrogen at 100 kPa. The low capacity should be ascribed to the large size of nanoparticles, and the long range of undecorated inner surfaces.

It is deduced that the key for hydrogen spillover is high Pt dispersion. We compared the dispersion behaviors of 5.0PtAS and 5.0PtCS samples with identical Pt content. In detail, for the 5.0PtAS sample, Pt nanoparticles highly dispersed with the size of 3.7 nm. For the 5.0PtCS sample, Pt nanoparticles sparsely dispersed with the large size of 7.7 nm. We also compared the hydrogen adsorption performance of the two samples. The amount of hydrogen captured can reach 0.42 mmol∙g^−1^ over 5.0PtAS, which is much higher than that over 5.0PtCS (0.21 mmol∙g^−1^). It is observed that the spillover effect is much greater in 5.0PtAS than in 5.0PtCS. The better hydrogen spillover effect was achieved when the particle size was 3.7 nm, and that worse spillover effect was detected when the size was as large as 7.7 nm. It is reasonable to verify that the small size of Pt nanoparticles is necessary to facilitate hydrogen spillover. Similar rules were found on the reported Pt/C materials for hydrogen spillover adsorption^53^.

In this work, we mainly researched the influence of Pt dispersion behaviors on the effect of hydrogen spillover. For this purpose, we only used the sole support of SBA-15. Besides, other factors may also have influence on the effect of hydrogen spillover. It is recently reported that the support has great influence on the effect of hydrogen spillover^54^. According to their results, hydrogen spillover is much faster on a “reducible” substrate than that on a “nonreducible” surface. In the future, we may carry out some researches on the aspects. Moreover, carbon bridges in the samples can assist hydrogen spillover^19^. The residual carbon content in resultant 5.0PtAS sample (0.99 wt%) is a little more than that in 5.0PtCS (0.19 wt%) (Table S1). Though the low carbon content in the two samples, the more content of residual carbon in the 5.0PtAS sample may be beneficial to the better capacity to some extent.

## Methods

### Materials synthesis

Mesoporous silica SBA-15 was synthesized according to the procedure reported by Zhao *et al*.^55^. 2 g of Pluronic P123 (EO_20_PO_70_EO_20_) was first dissolved in 75 g of HCl aqueous solution (1.6 M). Then 4.25 g of silica source tetraethylorthosilicate (TEOS) was added and stirred at 40 °C for 24 h, followed by hydrothermal treatment at 100 °C for 24 h. The as synthesized sample was recovered by filtration and dried at room temperature. The as synthesized SBA-15 was then calcined at 550 °C for 5 h in flowing air to generate template free SBA-15. Thermogravimetric (TG) analysis shows a weight loss of 50% below 600 °C, due to the decomposition of template. This is consistent with the reported value (52%), indicating that the template is maintained in the pores of as synthesized SBA-15^46, 56^.

The precursor of H_2_PtCl_6_·6H_2_O was introduced into as synthesized SBA-15 *via* solid-state grinding at ambient conditions for 20 min. The thoroughly mixed powder was calcined in flowing air at 300 °C for 2 h, and followed by reduction in 10% H_2_/Ar mixture gases at 200 °C for 2 h. The obtained materials were denoted as *x*PtAS, where *x* represented the content of Pt in the resultant samples (wt%). In a similar process, *x*PtCS samples were prepared by introduction of H_2_PtCl_6_·6H_2_O into template free SBA-15 for comparison.

### Characterization

X-ray diffraction (XRD) patterns of the meterials were recorded on a Bruker D8 Advance diffracto-meter with Cu *Kα* radiation in the 2*θ* ranges from 0.7° to 3° (for low-angle patterns) and 5° to 80° (for wide-angle patterns) at 40 kV and 40 mA. Transmission electron microscopy (TEM) was performed on a JEM-200CX electron microscope operated at 200 kV. The N_2_ adsorption-desorption isotherms were measured using a BELSORP-max system at −196 °C. Prior to analysis, the resultant SBA-15, PtAS, and PtCS samples were evacuated at 200 °C for 4 h. The as synthesized SBA-15 and 5.0PtAS samples without calcination were evacuated at 100 °C for 4 h. The Brunauer-Emmett-Teller (BET) surface area was calculated using the adsorption branch in the relative pressure range from 0.04 to 0.20. The total pore volume was derived from the amount adsorbed at the relative pressure of about 0.99. The pore size distribution was calculated by Barrett-Joyner-Halenda (BJH) method according to the adsorption branch.

Thermogravimetric (TG) analysis was performed on a thermobalance (STA-499C, NETZSCH). About 10 mg of sample was heated from the room temperature to 800 °C in a flow of air (25 mL·min^-1^). Fourier transform infrared (IR) spectra were recorded on a Nicolet Nexus 470 spectrometer with a spectra resolution of 2 cm^-1^ using transparent KBr pellets. Elemental analysis experiment was carried on Elementar Vario EL III instrument. Before analysis, the freshly ground 5.0PtAS samples were calcined in air at different temperatures for 2 h. The calcined samples were named as 5.0PtAS-T, where T represented the calcination temperature.

### Hydrogen adsorption test

Hydrogen adsorption isotherms were measured at 298 K using a volumetric analyzer (ASAP 2020) supplied by Micromeritics. Prior to measurement, the samples were pretreated under vacuum at the temperature of 200 °C for 4 h.

## Conclusions

In conclusion, by use of the nanoconfinement effects provided by as synthesized SBA-15, the Pt nanoparticles could be highly dispersed inside the channels of SBA-15. The size of the Pt nanoparticles is in control of 3.7 nm with the content of Pt loading up to 5.0 wt%. With combination of the controllable nanoparticle size and high loading content, the Pt nanoparticles could spread over the inner surface of SBA-15. And there is undecorated surface of SBA-15 surrounding each nanoparticle as well. The Pt nanoparticles work as active sites to dissociate hydrogen molecules. And the inner surface of SBA-15 works as the accepting surface to adsorb dissociated hydrogen atoms. The excellent coordination of Pt nanoparticles and SBA-15 endows the materials a great potential for hydrogen storage at room temperature.

## Electronic supplementary material

supplementary information
