# Supplementary material for: Modification of as Synthesized SBA-15 with Pt nanoparticles: Nanoconfinement Effects Give a Boost for Hydrogen Storage at Room Temperature
Source: Sci Rep. 2017 Jul 3;7:4509. doi: 10.1038/s41598-017-04346-9 (PMC5495762; doi:10.1038/s41598-017-04346-9)
Supplement: Supplementary file 1 — supplementary information [file 41598_2017_4346_MOESM1_ESM.doc]

**Supplementary Information**

**Modification of as Synthesized SBA-15 with Pt nanoparticles: Nanoconfinement Effects Give a Boost for Hydrogen Storage at Room Temperature**

Yu Yin, ^1^ Zhi-Feng Yang, ^1^ Zhi-Hao Wen, ^1^ Ai-Hua Yuan, ^1^ * Xiao-Qin Liu, ^2^ * Zhuang-Zhuang Zhang ^1^ and Hu Zhou ^3^

*^1^* *School of Environmental and Chemical Engineering,* *Jiangsu University of Science and Technology,* *Zhenjiang 212003, China*

*^2^ State Key Laboratory of Materials-Oriented Chemical Engineering, College of Chemistry and Chemical Engineering, Nanjing Tech University, Nanjing 210009, China*

*^3^* *School of Material Science and Engineering, Jiangsu University of Science and Technology, Zhenjiang 212003, China*

**Corresponding Authors.** [aihua.yuan@just.edu.cn](mailto:aihua.yuan@just.edu.cn); [liuxq@njtech.edu.cn](mailto:liuxq@njtech.edu.cn)

**Supplementary Experimental Details**

**Materials Characterization.**

High resolution transmission electron microscopy (HRTEM) was performed on a Tecnai G2 F30 S-Twin electron microscope operated at 300 kV.

Elemental analysis experiment was carried on Elementar Vario EL III instrument. The 5.0PtAS and 5.0PtCS samples were prepared with solid-state grinding, calcination in air, and H2/Ar gases.

**Supplementary Figures and Tables**

**Figure S1.** Low-angle XRD patterns for the samples of SBA-15, PtAS and PtCS.


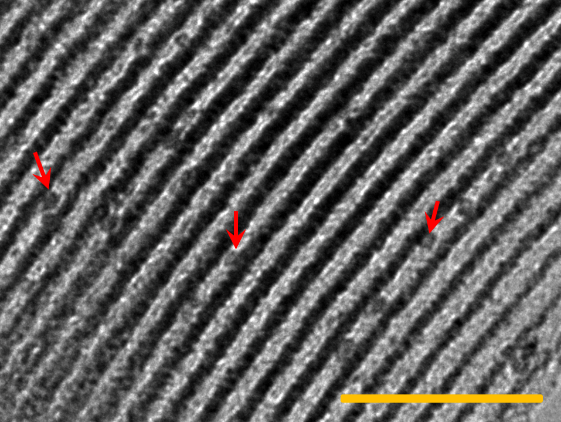


**Figure S2.** HRTEM images for the samples of 5.0PtAS. Scale bars represent 50 nm.

**Table S1.** Elemental analysis results of C and H weight ratio in the samples

| sample | C (wt %) | H (wt %) |
| --- | --- | --- |
| 5.0PtAS | 0.99 | 2.23 |
| 5.0PtCS | 0.19 | 1.58 |
